# Successful Integration of Hepatitis C Virus Point-of-Care Tests into the Denver Metro Health Clinic

**DOI:** 10.1155/2013/528904

**Published:** 2013-12-22

**Authors:** A. Jewett, A. A. Al-Tayyib, L. Ginnett, B. D. Smith

**Affiliations:** ^1^Oak Ridge Institute for Science and Education, 1600 Clifton Road, MS C-01, Atlanta, GA 30333, USA; ^2^Denver Public Health, Denver, CO 80204, USA; ^3^Department of Epidemiology, Colorado School of Public Health, Aurora, CO 80045, USA; ^4^Centers for Disease Control and Prevention, Division of Viral Hepatitis, Atlanta, GA 30329, USA

## Abstract

*Background*. The Centers for Disease Control and Prevention (CDC) recommends testing and linkage to care for persons most likely infected with hepatitis C virus (HCV), including persons with human immunodeficiency virus. We explored facilitators and barriers to integrating HCV point-of-care (POC) testing into standard operations at an urban STD clinic. *Methods*. The OraQuick HCV rapid antibody test was integrated at the Denver Metro Health Clinic (DMHC). All clients with at least one risk factor were offered the POC test. Research staff conducted interviews with clients (three HCV positive and nine HCV negative). Focus groups were conducted with triage staff, providers, and linkage-to-care counselors. *Results*. Clients were pleased with the ease of use and rapid return of results from the HCV POC test. Integrating the test into this setting required more time but was not overly burdensome. While counseling messages were clear to staff, clients retained little knowledge of hepatitis C infection or factors related to risk. Barriers to integrating the HCV POC test into clinic operations were loss to follow-up and access to care. *Conclusion*. DMHC successfully integrated HCV POC testing and piloted a HCV linkage-to-care program. Providing testing opportunities at STD clinics could increase identification of persons with HCV infection.

## 1. Background

Chronic hepatitis C virus (HCV) infection affects 3.2 million persons in the US [1] and approximately 45%–85% of infected persons are unaware of their infection [2]. The Centers for Disease Control and Prevention (CDC) recommends HCV testing for those persons born during 1945–1965 [2] and those most likely to be infected with HCV [3], including persons with human immunodeficiency virus (HIV) [4, 5]. Persons with HIV are disproportionately affected, with 25%–30% of HIV-infected persons being coinfected with HCV [6]. The CDC further recommends linkage to care (LTC) and treatment as appropriate for individuals with confirmed infection. Sexually transmitted disease (STD) clinics are examples of integrated healthcare facilities that provide a broad range of healthcare services (i.e., screening and testing, linkage to care) to clients in need of insurance assistance, charitable care, and/or anonymity. CDC's 2010 STD treatment guidelines recommend routine HCV testing of HIV-infected persons [7]. This recommendation suggests that clinics that reach persons at high risk for HIV infection may also reach HCV-infected persons. Prior to the CDC recommendation, one study from the mid-2000s showed only 54% of HIV-infected clients were screened for HCV in STD clinics [8].

The most commonly used tests to identify HCV antibodies are enzyme immunoassays (EIAs). EIAs require phlebotomy and laboratory analysis of specimens involving 1-2 weeks to receive test results. In February 2011 the United States Food and Drug Administration approved the OraQuick HCV Rapid Antibody Test for use with fingerstick whole blood samples. This point-of-care (POC) test provides results in 20 minutes [9–11].

Given the recent recommendations from the CDC and United States Preventive Services Task Force [12] to test all persons born during 1945–1965 and persons at high risk of infection, we expect demand for HCV testing to increase. In anticipation of expanded HCV testing, we conducted a demonstration study to explore the facilitators and barriers to integrating HCV POC testing into standard clinic operations at a busy, urban, STD clinic.

## 2. Methods

### 2.1. Setting

The Denver Metro Health Clinic (DMHC) is the largest STD clinic and HIV testing facility in the Rocky Mountain region with approximately 18,000 visits annually. The clinic offers an array of services including free and confidential STD and HIV testing, counseling, and treatment, as well as family planning and immunization services. Since 2000, DMHC has offered HCV testing using the standard HCV EIA test, though the volume of testing has dramatically decreased since 2005 due to funding and the barriers to follow-up for those identified as antibody positive. The DMHC is the STD clinic at Denver Public Health (DPH), the local health department for the City and County of Denver, and is an integrated part of Denver Health, Colorado's primary “safety net” institution. As part of a collaborative demonstration project between CDC and DPH a protocol was developed to integrate the OraQuick HCV Rapid Antibody Test and quantitative polymerase chain reaction testing (PCR) to establish viremia for those with a positive POC test, into clinic operations at the DMHC.

#### 2.1.1. Testing and Counseling Protocol

Prior to offering the HCV POC test in the clinic, DMHC staff participated in several planning meetings to elicit feedback on how best to integrate the HCV POC test into standard clinic procedures. Clinic staff received education and training related to hepatitis C. Specifically, staff received education about the epidemiology and pathogenesis of HCV, methods of transmission, complications related to HCV infection, diagnosis and testing, current treatment options, and contraindications for treatment. In addition, staff received education about ways to potentially reduce morbidity and mortality related to hepatitis C (e.g., avoiding alcohol, getting vaccinated for hepatitis A and B, etc.). Furthermore, staff contributed to the development of prevention messages for clinic clients. Development of the protocol was an iterative process during which feedback from clinic staff was incorporated into the protocol and staff participated in role playing to refine the prevention messages.

Between June 4, 2012 and September 29, 2012, approximately 3,000 persons presenting to the DMHC were asked a set of screening questions by triage staff to determine if they had risk factors for HCV infection. Screening questions included date of most recent injection, ever receiving a tattoo in an unregulated facility, ever sharing equipment for intranasal drug use, ever having sex with a HCV-positive partner, receiving blood or blood products prior to 1992, and birth year during 1945–1965 (see Appendix A for complete listing of triage eligibility questions). An HCV POC test was offered to 926 clients who reported at least one risk factor.

Following triage, 50 (5.4%) clients refused to be tested and the 876 who agreed to the HCV POC test were taken to a phlebotomy station where two tubes of blood were drawn. One tube was used for the POC HCV test and a rapid HIV test if the client had not received an HIV test in the past 12 months. The second tube was used for the confirmatory PCR HCV RNA test if needed. Though the HCV POC test can be performed on a fingerstick blood sample, drawing blood was the most efficient way to integrate the HCV POC test since HIV testing is conducted via phlebotomy as well. Once specimens were drawn they were immediately sent to the on-site laboratory which provided the HCV result in 20 minutes. The laboratory staff and clinic staff access the same electronic medical record making results available to the provider to give to the client at the end of the clinic visit. A total of 33 anti-HCV positive POC tests were observed and 32 specimens were then reflexed for a quantitative PCR HCV RNA test. Of the 32 specimens tested for HCV RNA, 21 were HCV RNA positive indicating current viral infections. The anti-HCV POC negative rapid test result specimens were discarded and attempts were made to give results to clients. No other follow-up was conducted with anti-HCV negative clients.

While demographic data were not collected as part of this study, in 2013, the year following our data collection period, the clinic population screened with the HCV POC test was 74% male, had a median age of 30 (IQR: 24,42), and 47% reported being white non-Hispanic, 17% black non-Hispanic, and 29% Hispanic. Given the small number of staff members who were interviewed, demographic data are not reported to maintain their confidentiality.

All clients were provided with educational and prevention messages. Providers presented risk reduction messages and strategies to clients with a HCV-negative POC test result based on the client's identified risk factor. Clients with a positive test were provided with a more extensive counseling session with linkage-to-care (LTC) counselors. During the session, LTC counselors provided clients with information about HCV including differences between acute and chronic HCV, the difference between screening and confirmatory tests and what each means, percentage of people who clear the infection and what that means, and what hepatitis does to the liver and body. In addition, clients were provided information about how to prevent transmitting HCV to others, were counseled on alcohol and drug avoidance, offered hepatitis A and B vaccinations, provided with general information about treatment, and were scheduled for an appointment to receive their confirmatory HCV RNA result. DPH used the demonstration study as an opportunity to develop and pilot a LTC protocol for HCV utilizing existing HIV LTC counselors (see Appendix B for flow diagram of HCV POC demonstration program).

The research associated with the integration of the HCV POC test into clinic operations involved focus groups and interviews with clinic staff and clients who participated in the process. The study was determined to be nonresearch by CDC and received approval from the Colorado Multiple Institutional Review Board.

### 2.2. Interviews with Clients

Clients who received the HCV POC test were recruited in the clinic to provide feedback on their testing and counseling experiences. Research staff conducted interviews with only three HCV-positive clients and nine HCV-negative clients. These small samples were primarily due to the absence of current contact information or rapid change in contact information given the somewhat transient housing situations many of the clinic's clients have. Clients who agreed to participate in the interview but asked to schedule it for a later time were difficult to reach. Interviews lasted approximately 15 minutes. Clients were asked about their knowledge of hepatitis C and HCV testing, their feelings about the screening questions, about facilitators and barriers to getting tested, and any messages received during counseling (Appendix C).

### 2.3. Focus Groups with Staff

Two focus groups with a total of 19 participants were conducted with triage staff, laboratory staff, health care providers (i.e., RNs, LPNs, nurse practitioner, and health care partners), and LTC counselors, which lasted between 15 and 30 minutes. Staff were asked about their knowledge of hepatitis C, HCV testing, and HCV counseling. Additionally, they were asked about barriers and facilitators of HCV testing and counseling, as well as their suggestions for promotional ideas (Appendix D).

### 2.4. Qualitative Analysis

Interviews were recorded and transcribed. Data coding and quality assurance was performed by a multidisciplinary team including a research analyst, principal investigator, and CDC project officer using Microsoft Excel 2010. Based on Grounded Theory [13], themes and subthemes were identified and validated by peer triangulation. For ease of use and clarity, themes and results are presented by participant group (i.e., HCV-negative clients, HCV-positive clients, triage staff, health care providers, and LTC staff).

## 3. Results

### 3.1. HCV-Negative Clients

Overall, the nine clients with a HCV-negative POC test result (1.1% of all persons who tested anti-HCV negative) were pleased with the process of testing and counseling. Clients reported liking that the test was rapid, that it was conducted in conjunction with other tests, and that they received their results the same day. When interviewees were asked about the screening questions and how the sensitive questions made them feel, most felt they were necessary. However, only two interviewees reported that the screener made them aware of risk factors for hepatitis C.

The counseling messages provided by triage staff had limited success in conveying to clients an understanding about HCV transmission, diagnostics, and treatment. Most clients correctly understood that hepatitis C was transmitted via blood, but a smaller number of interviewees understood that transfusions (3 clients) and injection drug use or needles (5 clients) are vehicles for transmission. Most HCV-negative clients did not have a clear understanding of the POC hepatitis C antibody test: one stated that it provided rapid results; one stated it was an antibody test; the others reported not understanding or were uncertain about their understanding. Three interviewees incorrectly reported that hepatitis C was incurable. All HCV-negative respondents reported the knowledge gained and/or learning their status was a benefit of testing, with two reporting that it made them aware of the risk factors for hepatitis C. HCV-negative clients reported having few fears about being tested. The most frequently noted fear was the potential to receive a positive result (5 clients), while three clients reported feeling fear of the phlebotomy needle or having blood drawn.

The counseling messages presented to HCV-negative clients by providers also had limited success. Most clients understood a POC test message, with six clients reporting that the result would be returned quickly, but clients did not retain any details about the test. Risk factor messaging was also limited in success. Although all nine clients had a risk factor, only four interviewees accurately reported the risk factor that triggered their own testing.

HCV-negative clients were asked about the prevention messages they received from providers. Most respondents heard messages about needles, but understood them inconsistently. Four reported hearing that they should not use needles or intravenous drugs, six stated “do not share needles” and one stated “do not share needles except for insulin or something.” Other prevention messages reported by clients included: “do not get unprofessional tattoos” (5), “have safe sex” (1), and “do not share personal hygiene items” and “clean surfaces with blood spills” (1).

### 3.2. HCV-Positive Clients

The three HCV POC test positive clients (9.1% of all persons who tested anti-HCV positive) were more consistent in terms of knowledge acquired than HCV-negative clients. All three reported knowing that hepatitis C was a virus and that it affected the liver. All HCV-positive interviewees also reported knowledge gain as a benefit of counseling and testing. Learning about hepatitis C and available treatment options was also beneficial to clients. Clients understood that the POC HCV test is a blood test and one reported it was an antibody test. Two clients reported that the process of testing was “rapid.”

When clients were asked about the process and what made it easier for them to get tested, one reported knowing the counselor would help them, one reported that it was quick, and one reported that they could receive all their testing at once. All three interviewees reported not feeling ashamed when asked screening questions regarding their risk factors.

HCV-positive clients remembered messages that were given to them by the counselors. Two reported that alcohol could make their condition worse, two said they were encouraged to see a specialist or get treatment, and one remembered that a positive rapid test does not necessarily mean they are infected.

Clients noted that knowing the truth about one's HCV status, knowing the risk factors, and understanding the disease were messages that should be promoted to increase rapid HCV testing. Risk factors reported by these clients included injection drug use (2), sexual contact (2), and receiving a tattoo (1). When asked about the prevention messages they received, two clients stated not to share injection equipment, one stated to use condoms, and one said no blood-to-blood contact.

### 3.3. Public Health Clinic Staff

Clinic staff participated in training on the disease, testing, counseling, and treatment options. Triage counselors, providers, and LTC counselors were involved in the testing process and participated in focus groups. 

#### 3.3.1. Triage Staff

Triage staff reported being somewhat familiar with hepatitis C before the project. During training, staff increased their knowledge of hepatitis C testing as well as POC tests in general. Triage staff reported that asking clients the screening questions was not difficult. However, staff reported that screening during triage should include questions regarding injection and sexual partners with hepatitis C.

The process of integrating POC testing into their current setting required more time, but staff indicated that it was not overly burdensome. Triage staff reported that the HCV POC test was quick, accurate, and simple to use. Barriers for integrating the test into their workload included forgetting to draw an additional tube of blood and adding a few minutes to a timed visit. However, the staff reported that increasing their knowledge and familiarity with the process allowed them to overcome these obstacles with relative ease.

#### 3.3.2. Health Care Providers

Providers reported the counseling messages to be delivered to the clients were clear and specific. They also reported that the messaging did not contradict any of the other messages that were given, specifically regarding HIV or other STDs. Problems encountered by providers included that clients had many questions, clients incorrectly believed they had received the test before (assuming it was a standard test conducted at annual visits), and clients provided different information to different staff within the clinic. Providers reported the most difficult questions asked by clients included “am I going to die?” and “how do I tell my partner?” When the providers were asked what messages should be used to promote the HCV POC test, they indicated “same-day results,” having a hepatitis clinic (within the public health clinic), and “there is someone to help with care and next steps.”

#### 3.3.3. LTC Counselors

Prior to this study, LTC counselors' experience in hepatitis C and hepatitis C counseling varied from very little to 10 years of experience. Similar to the providers, the LTC counselors felt the messages were clear and specific. Barriers identified included being unable to answer all of the clients' questions, loss to follow-up, and finding resources for care. Questions that were difficult to answer were “am I going to die?”, “will I get sick?”, “can I give it to someone else?”, and “can I get financial assistance?” LTC counselors reported that some HCV-positive clients were difficult to locate or the clients did not provide accurate contact information and therefore were considered lost to follow-up. The LTC counselors found it difficult to identify resources for hepatitis C clients. They were unable to tap into previously identified HIV resources because those resources were not available to HCV-positive clients. The LTC counselors agreed with triage staff that although it added to their workload, the hepatitis C counseling was not too burdensome.

## 4. Discussion

Denver Public Health successfully integrated rapid HCV testing into the DMHC. In addition, DPH successfully developed and piloted an HCV linkage-to-care program. One facilitator of this success was the reduction in the amount of time required for clients to receive results when using the HCV POC in comparison with EIA testing, which requires a subsequent visit to receive results [14]. Using the HCV POC test, staff had the opportunity to educate clients about their potential risks for HCV acquisition and the consequences of HCV infection. Providing clients with results at the point of care also allowed for an immediate response from the LTC counselors to inform positive clients about the process of receiving medical evaluation and treatment and care, as appropriate.

A second facilitator of success was the ability to evaluate individuals who presented to the clinic in a systematic way to assess the need for the HCV POC test based on their reported risk. Providing information about HCV and potential risk for HCV infection during the triage process helped facilitate testing in addition to providing an opportunity to increase awareness of HCV. While the triage questions were successful in identifying the need for a HCV POC test, one question related to intranasal drug use required an adjustment. During 8 days following implementation, the question “when was the last time you used intranasal drugs?” was used as an indicator of risk. During the 8 days, 134 people were screened, 94 reported intranasal drug use, and 61 of those reported that this was their only risk factor. All 61 were HCV negative. To clarify the risk based on blood exposure, “did you share a dollar bill or straw to do so?” was added.

A notable concern is the inconsistent understanding of HCV that clients reported after counseling, despite the perception of staff that the messages were clear. The apparent disconnect between the messages staff deliver and the messages clients receive highlights the need to engage all stakeholders, including staff and clients, in the development of counseling messages.

Education of the staff prior to the beginning of the project was an essential component that contributed to successful integration of the HCV POC test. Initial and ongoing HCV training to clinic staff increased knowledge about the virus, how it is transmitted, and the importance of diagnosing individuals who are infected. Although this knowledge was helpful in communicating with clients, many client questions that fell outside the scope of testing (i.e., am I going to die) were challenging to manage. Staff also reported challenges in accessing resources for HCV-infected clients. Clinic staff better understood the need for HCV testing when presented with data highlighting the scope of the problem. Finally, demonstrating to clinic staff that the HCV POC test could be easily incorporated into the daily routine allowed them to successfully manage the limited additional work that was required.

There are a number of lessons learned from this study. (1) Involving all stakeholders in the development and implementation of the project is essential for success. (2) Engaging the target population in the development of educational and prevention messages should increase the likelihood that these messages will be heard. (3) Providing an orientation and education about the testing effort to staff will ease implementation and contribute to the success of the initiative. It also should be noted that time would be well spent by engaging potential clients before the beginning of the testing effort to better inform staff about the type of questions that may be asked so they can be better prepared to manage these queries. Lastly, while resources may be a challenge, the needs of persons who are infected should be addressed before a testing effort begins to ensure that clients will have access to support.

DMHC is part of a large, integrated public health system and therefore our experience is not generalizable to all public health clinics. Additionally, we conducted only three interviews with HCV positive clients because the clientele are difficult to reach for post appointment follow-up; this emphasizes the need for POC testing, counseling, and LTC programs for HCV positive clients. Providing testing opportunities at STD clinics could increase the identification of HCV infection among persons at increased risk such as those born from 1945 to 1965, those with HIV infection, and those with a history of injection drug use. Integration of HCV POC testing should be considered in this type of setting based on ease of use, limited additional workload, and direct benefits for clients in terms of knowing their status, preventing transmission to others, and managing disease for those who are infected.

## Figures and Tables

**Figure 1:**
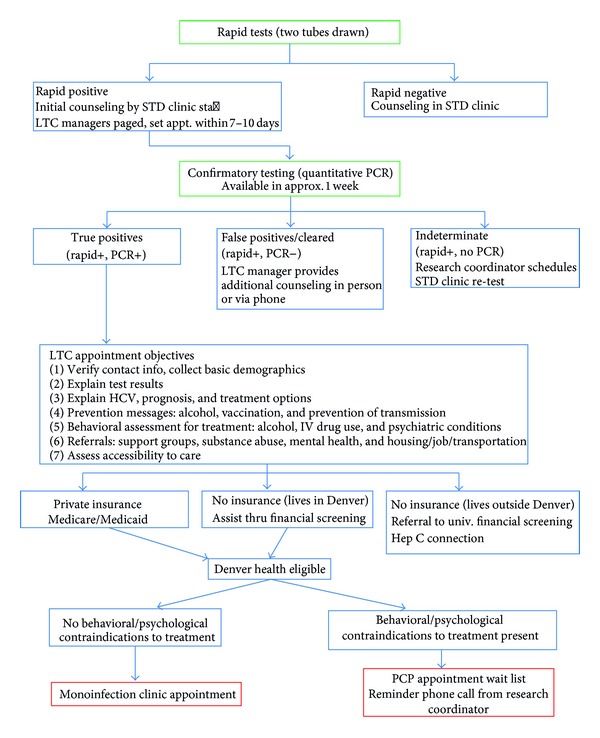
HCV POC Demonstration Project Protocol at Denver Public Health.

## Appendices

### A. Rapid Hepatitis C Test Triage Eligibility Questions

When was the last time you tested for the hepatitis C virus (HCV)?

Hepatitis C is a viral infection passed by contact with an infected person's blood. It does not take much blood to pass the virus. Many people with HCV do not know that they have it, and the only way to know is through a test. There is no vaccine for HCV. I am going to ask you some questions about things that may put you at risk for HCV.

When was the last time you used needles to inject drugs, which also includes steroids (not counting insulin)?

HCV can be transmitted through shared syringes, cottons, cookers, rinse water, and tourniquets that are used in the process of injecting drugs or steroids.

When was the last time you used intranasal drugs (i.e., snorted drugs), legal or illegal? Did you share a straw or dollar bill to do so?

Mucosal membranes in the nose are very sensitive and can easily bleed while snorting drugs. If a straw or dollar bill is shared to snort drugs, HCV can be transmitted through small amounts of blood on the straw or dollar bill.

Have you ever received a tattoo in an unprofessional environment (prison, home)?

Tattooing supplies that are not properly sterilized (such as in a professional setting) can transmit HCV. Tattoo ink, if shared, can also transmit HCV.

Have you ever had a sexual partner who is HCV positive?

Although unlikely, HCV can be transmitted through unprotected sex if blood is present. The chances of transmitting HCV this way increase with additional sexual partners, with rough sex, and if other STDs are present.

Have you ever received blood or blood products, like a transfusion, before 1992 or outside of the United States?

The blood supply in the U.S. was not tested for HCV until 1992. Many countries still do not test their blood supply for HCV, so receiving blood or blood products in another county at any time could be a risk factor for contracting HCV.

Were you born between 1945 and 1965?

Baby boomers have been shown to have a much higher percentage of HCV than other populations. This could be due to many factors, but is mainly because of risk behaviors that occurred over 30 years ago (i.e., drug use, risky sexual practices, tattoos or blood transfusions in other countries, military service, etc.).

If the client answers “yes” to any of the questions, the client is eligible for HCV rapid testing.

Circle the HCV test on the registration form and complete the daily tally sheet.

### B.

See Figure 1.

### C. HCV Demonstration Project Formative Evaluation

#### C.1. Individual Interview Questions

1. Before Today
  - Have you ever been tested for hepatitis C?
  - Have you ever been told you had hepatitis C?
  - Based on your appointment today, what is your understanding of hepatitis C?
  - What is your understanding of the hepatitis C antibody test you were given today?
2. Getting Tested
  - What do you think about the HCV rapid test process?
  - What about the process made it easier for you to get tested?
  - What about the process made it harder for you to get tested?
  - What would you describe as fears about getting tested?
  - What would you describe as problems about getting tested?
  - What benefits did you experience?
3. Messaging
  - How did you feel about the hepatitis C screening questions you were asked today regarding your risk factors for hepatitis C?
  - What were you told about getting tested?
  - What do you remember about testing?
  - What messages did you hear?
4. If you were charged with promoting hepatitis C testing, what would you say or do?
5. What risk factors for hepatitis C do you feel you have?
  - If Injecting Drugs
    - What is your drug of choice?
    - How do you prepare your drugs?
    - How do you use your drugs?
    - Do you share supplies to inject your drugs (syringes, works, etc.)?
  - If Snorting Drugs
    - What is your drug of choice?
    - How do you prepare your drugs?
    - How do you use your drugs?
    - Do you share supplies to inject your drugs (straw or dollar bill)?
6. What was the result of your hepatitis C test today?
  - If Negative
    - Can you explain how to prevent contracting hepatitis C?
  - If Positive
    - Can you explain how to prevent transmitting hepatitis C to others?
    - Did you meet with a Linkage-to-Care counselor today?

### D. HCV Demonstration Project Formative Evaluation

#### D.1. Focus Group Questions

*Triage*

1. Background
  - Were you familiar with hepatitis C before this project?
2. Triage Questions
  - Were the triage questions appropriate?
  - Were the triage questions easy to ask?
3. Integration
  - What interfered with integrating this test into your workload?
4. If you were charged with promoting the HCV rapid test, what would you say or do?

*Testers*

1. Background
  - Have you ever conducted a hepatitis C test before?
  - Have you ever conducted a rapid test before?
2. The Rapid Test
  - What did you like about the hepatitis C rapid test?
  - What problems did you have conducting the test?
3. Integration
  - What interfered with integrating this test into your workload?
4. If you were charged with promoting the HCV rapid test, what would you say or do?

*Counselors*

1. Background
  - Do you have prior experience with hepatitis C counseling?
  - Do you have prior experience with rapid test counseling?
2. Message Delivery
  - What did you like about the messaging?
  - What problems did you encounter?
  - What questions did clients ask that were difficult to answer?
  - How did the HCV messages impact other messages?
3. If you were charged with promotion of the HCV rapid tests, what would you say or do?

*Linkage to Care*

1. Background
  - Were you familiar with hepatitis C before this project?
  - Do you have prior experience with hepatitis C counseling?
  - Do you have prior experience with rapid test counseling?
2. Message Delivery
  - What did you like about the messaging?
  - What problems did you encounter?
  - What questions did clients ask that were difficult to answer?
3. Integration
  - What interfered with integrating this counseling into your workload?
4. If you were charged with promoting the HCV rapid test, what would you say or do?

## References

[1] Armstrong GL, Wasley A, Simard EP, McQuillan GM, Kuhnert WL, Alter MJ (2006). The prevalence of hepatitis C virus infection in the United States, 1999 through 2002. *Annals of Internal Medicine*.

[2] Smith BD, Morgan RL, Beckett GA (2012). Recommendations for the identification of chronic hepatitis C virus infection among persons born during 1945–1965. *Morbidity and Mortality Weekly Report*.

[3] Recommendations for prevention and control of hepatitis C virus (HCV) infection and HCV-related chronic disease (1998). Centers for Disease Control and Prevention. *Morbidity and Mortality Weekly Report*.

[4] 1999 USPHS/IDSA guidelines for the prevention of opportunistic infections in persons infected with human immunodeficiency virus (1999). U.S. Public Health Service (USPHS) and Infectious Diseases Society of America (IDSA). *Morbidity and Mortality Weekly Report*.

[5] Kaplan JE, Masur H, Holmes KK (2002). Guidelines for preventing opportunistic infections among HIV-infected persons—2002. Recommendations of the U.S. Public Health Service and the Infectious Diseases Society of America. *Morbidity and Mortality Weekly Report*.

[6] Alter MJ (2006). Epidemiology of viral hepatitis and HIV co-infection. *Journal of Hepatology*.

[7] Workowski KA, Berman S (2010). Sexually transmitted diseases treatment guidelines, 2010. *Morbidity and Mortality Weekly Report*.

[8] Hoover KW, Butler M, Workowski KA (2012). Low rates of hepatitis screening and vaccination of HIV-infected MSM in HIV clinics. *Sexually Transmitted Diseases*.

[9] Services UDoHaH (2010). *FDA Approves Rapid Test For Antibodies To Hepatitis C Virus*.

[10] Lee SR, Kardos KW, Schiff E (2011). Evaluation of a new, rapid test for detecting HCV infection, suitable for use with blood or oral fluid. *Journal of Virological Methods*.

[11] Smith BD, Drobeniuc J, Jewett A (2011). Evaluation of three rapid screening assays for detection of antibodies to hepatitis C virus. *Journal of Infectious Diseases*.

[12] Moyer VA (2013). Screening for hepatitis C virus infection in adults: U.S. preventive services task force recommendation statement. *Annals of Internal Medicine*.

[13] Strauss ACJ (1990). *Basics of Qualitative Research: Grounded Theory Procedures and Techniques*.

[14] Smith BD, Teshale E, Jewett A (2011). Performance of premarket rapid hepatitis C virus antibody assays in 4 national human immunodeficiency virus behavioral surveillance system sites. *Clinical Infectious Diseases*.

